# A rare widespread vasculitis with giant cells in small- and medium-sized arteries and veins

**DOI:** 10.1016/j.ero.2026.03.015

**Published:** 2026-04-18

**Authors:** Tomoaki Saito, Saeko Yamada, Remi Kawashima, Nanami Mino, Yoichi Yasunaga, Haruka Tsuchiya, Tetsuo Ushiku, Keishi Fujio

**Affiliations:** 1 Department of Allergy and Rheumatology, The University of Tokyo Hospital, Tokyo, Japan; 2 Department of Pathology, The University of Tokyo Hospital, Tokyo, Japan

A woman in her 70s presented with low-grade fevers and painful subcutaneous nodules on both thighs 1 year ago. A skin biopsy revealed an inflammatory cell infiltrate with fibrin thrombi in small arteries. A tentative diagnosis of polyarteritis nodosa was made. Contrast-enhanced computed tomography (CT) showed no vascular inflammation. She was treated with diaphenylsulfone, after which the rash resolved and C-reactive protein (CRP) fell from 16.5 to 0.5 mg/dL.

The fevers recurred 9 months later, and CRP increased to 4.8 mg/dL. Contrast-enhanced CT revealed extensive thickening of the aortic wall from the root to the common iliac arteries and its branches (Fig, A) with angiostenosis at the confluence of the superior mesenteric and splenic veins (Fig, B). Multiple pulmonary nodules near the interlobar fissure were noted (Fig, C,D) with increased uptake on positron emission tomography-CT in these areas (Fig, E-G). A lung biopsy revealed large epithelioid cell granulomas invading veins and the pleura, which were recognised as nodules on imaging, and vasculitis with giant cells in small- and medium-sized arteries and veins (Fig, H-J). The pulmonary specimen culture was negative, and Ziehl–Neelsen, Periodic Acid–Schiff, and Grocott staining revealed no microorganisms. Blood cultures for bacteria and acid-fast bacilli were negative, and haematologic tests excluded tuberculosis and viral infections.

**Figure:**
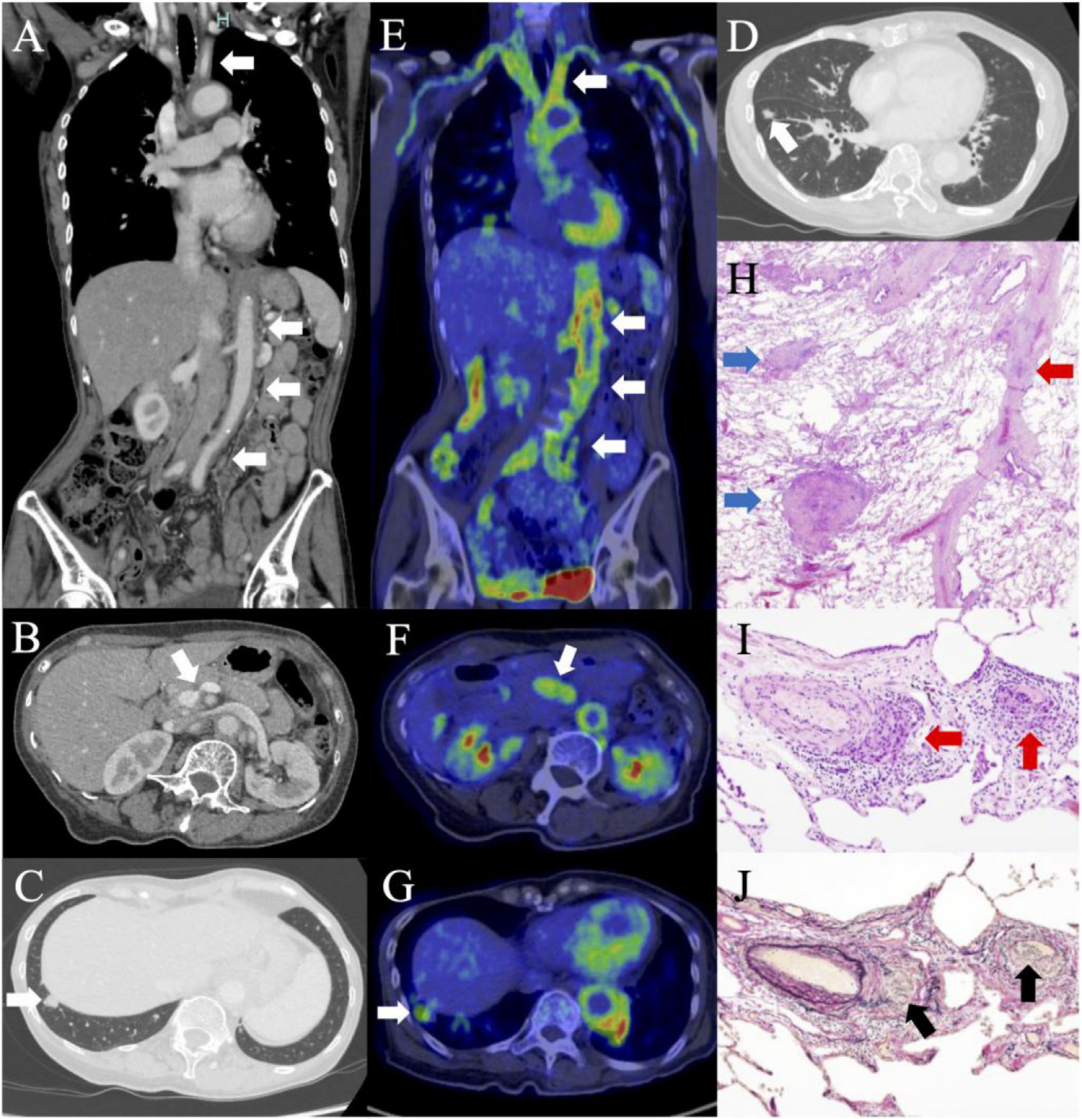
A, Extensive aortic wall thickening. B, Angiostenosis at the confluence of the superior mesenteric and splenic veins. C, D, CT showing multiple pulmonary nodules. E-G, PET-CT showing tracer accumulation in arterial lesions, venous lesions, and multiple pulmonary nodules. H-J, Lung biopsy showing large epithelioid cell granulomas invading veins (blue arrows) and the pleura, and vasculitis with giant cells in small- and medium-sized arteries (red arrows) and veins (H, I, H&E staining). The internal and external elastic laminae of small-sized arteries were disrupted (black arrows) (J, Elastica van Gieson staining of the same field of view as panel I). H, × 2; I, J, × 20. CT, computed tomography; H&E, hematoxylin and eosin; PET, positron emission tomography.

Infectious aortitis was excluded based on negative tests. Behçet’s disease was ruled out given a lack of characteristic symptoms. Sarcoidosis was considered unlikely because the granulomas were unusually large and showed prominent vascular involvement. Although necrotising sarcoid granulomatosis can present with necrotising granulomas and vascular involvement, the granulomas in this case frequently contained collagenous-appearing internal areas, which differed from the typical coagulative necrosis seen in necrotising sarcoid granulomatosis. Furthermore, lymphoma was excluded pathologically.

Although metformin, taken for decades in this case, can cause drug-induced vasculitis [1], the latency in our case was inconsistent with previous reports, which described a latency period ranging from 5 days to 4 months after the metformin initiation. In addition, there was no medical exposure to cyanoacrylate glue [2].

Overall, we suggested unclassified granulomatous vasculitis with arterial and venous involvement and concomitant large-vessel inflammation because these findings were inconsistent with known diseases. Prednisolone (1 mg/kg/d) and tocilizumab improve aortic wall thickening, pulmonary nodules, and venous stenosis on follow-up imaging 2 weeks later, with CRP decreasing from 7.95 to 0.05 mg/dL.

This case was distinguished from giant cell phlebitis [3] by the findings that both arteries and veins were involved and responded to immunosuppressants. Further case studies are warranted.

## Contributors

TS wrote the first manuscript with critical inputs from SY. TS, SY, RK, NM, YY and TU primarily conducted the medical treatment of this case. SY, HT and KF were involved in drafting the article or revising it critically for important intellectual content. All authors contributed to the final version of the manuscript and approved the final version for submission.

## Funding

This research did not receive any specific grant from funding agencies in the public, commercial, or not-for-profit sectors.

## Competing interests

TS has received speaking fees from Asahi Kasei Pharma Corporation and AstraZeneca K.K. SY has received speaking fees from Asahi Kasei Pharma Corporation, Pfizer, Bristol Myers Squibb, AstraZeneca K.K., Taisho Pharmaceutical, Eisai, and Eli Lilly. HT has received speaking fees and/or honoraria from AbbVie, Amgen, Asahi Kasei, Astellas, Bristol Myers Squibb, Chugai, Daiichi-Sankyo, Eisai, Eli Lilly, Gilead, Jansen, Novartis, Sanofi, Tanabe Mitsubishi, and UCB and research grants from AbbVie, Mochida, and Takeda. KF received research grants from Chugai Pharmaceutical Co, Ltd, AbbVie GK, Asahi Kasei Pharma Co, Ltd, Bristol Myers Squibb K.K., AstraZeneca K.K., Eisai Co, Ltd, Tsumura Co, Ltd, and Taisho Pharmaceutical Co, Ltd; consulting fees from Asahi Kasei Pharma Co, Ltd; payments or honoraria from Chugai Pharmaceutical Co, Ltd, AbbVie GK, Asahi Kasei Pharma Co, Ltd, Bristol Myers Squibb K.K., AstraZeneca K.K., Mitsubishi Tanabe Pharma Corporation, Eisai Co, Ltd, Gilead Sciences K.K., Eli Lilly Japan K.K., Pfizer Japan Inc, Taisho Pharmaceutical Co, Ltd, Astellas Pharma Inc, Daiichi-Sankyo Co, Ltd, Novartis Pharma K.K., GlaxoSmithKline K.K., Otsuka Pharmaceutical Co, Ltd, and Alexion Pharma GK, and is a member of an advisory board for Asahi Kasei Pharma Co, Ltd. All the other authors declare they have no competing interests.
